# Berry Fruit Consumption and Metabolic Syndrome

**DOI:** 10.3390/antiox5040034

**Published:** 2016-09-30

**Authors:** Stefano Vendrame, Cristian Del Bo’, Salvatore Ciappellano, Patrizia Riso, Dorothy Klimis-Zacas

**Affiliations:** 1 School of Food and Agriculture, Food Science and Human Nutrition, University of Maine, Orono, ME 04469, USA; stefano.vendrame@fulbrightmail.org; 2 DeFENS—Department of Food, Environmental and Nutritional Sciences, Division of Human Nutrition, Università degli Studi di Milano, 20133 Milan, Italy; cristian.delbo@unimi.it (C.D.B.); salvatore.ciappellano@unimi.it (S.C.); patrizia.riso@unimi.it (P.R.)

**Keywords:** berries, Metabolic Syndrome, dietary intervention studies, humans

## Abstract

Metabolic Syndrome is a cluster of risk factors which often includes central obesity, dyslipidemia, insulin resistance, glucose intolerance, hypertension, endothelial dysfunction, as well as a pro-inflammatory, pro-oxidant, and pro-thrombotic environment. This leads to a dramatically increased risk of developing type II diabetes mellitus and cardiovascular disease, which is the leading cause of death both in the United States and worldwide. Increasing evidence suggests that berry fruit consumption has a significant potential in the prevention and treatment of most risk factors associated with Metabolic Syndrome and its cardiovascular complications in the human population. This is likely due to the presence of polyphenols with known antioxidant and anti-inflammatory effects, such as anthocyanins and/or phenolic acids. The present review summarizes the findings of recent dietary interventions with berry fruits on human subjects with or at risk of Metabolic Syndrome. It also discusses the potential role of berries as part of a dietary strategy which could greatly reduce the need for pharmacotherapy, associated with potentially deleterious side effects and constituting a considerable financial burden.

## 1. Metabolic Syndrome: General Overview

Metabolic Syndrome is characterized by the simultaneous presence of multiple risk factors, which are a direct or indirect consequence of both insulin resistance and overweight/obesity [1]. Although it is not a disease in itself, this combination of health problems dramatically increases the risk of developing type II diabetes mellitus and cardiovascular disease [2].

The US National Cholesterol Education Program–Adult Treatment Panel III (ATPIII) defines Metabolic Syndrome as the combined occurrence of at least three of the following five risk factors: abdominal obesity (waist circumference ≥102 cm in males, ≥88 cm in females); high blood triglycerides (≥150 mg/dL); low HDL cholesterol (≤40 mg/dL in males, ≤50 mg/dL in females); high diastolic and/or systolic blood pressure (≥130/85 mmHg) and high fasting blood glucose (≥100 mg/L) [2].

Together with these diagnostic parameters, which were selected because of their widespread use in the clinical setting, a plethora of other dysfunctional states are often associated with Metabolic Syndrome. Although they are not used for diagnostic purposes, they significantly contribute to the increased cardiovascular risk and the onset of type II diabetes mellitus [2]. In particular, Metabolic Syndrome is associated with a pro-oxidant, pro-inflammatory, and pro-thrombotic state [3,4].

Chronic, low-grade, systemic inflammation is one of the landmark characteristics of Metabolic Syndrome, leading to unnecessary tissue damage, endothelial dysfunction, thrombosis, insulin resistance, high blood pressure, and all the pathologies related to such risk factors, including cardiovascular disease, diabetes, some forms of cancer, arthritis, neurodegenerative diseases, and many others [3,4].

Fat accumulation and obesity are major underlying causes of chronic inflammation, due to the inherent ability of adipocytes to secrete inflammatory mediators: mainly adipocyte-derived cytokines (called adipokines), but also hormones like leptin [5].

Inflammation and fat accumulation both lead to impairment in glucose metabolism and insulin resistance, and in turn, impaired glucose metabolism exacerbates inflammation and tissue damage, thus sustaining a pro-inflammatory state and endothelial dysfunction [6,7].

Indeed, another landmark characteristic of Metabolic Syndrome is the development of endothelial dysfunction, which is strictly related to oxidative stress and inflammation via the nuclear factor kappa-b pathway [1].

Endothelial dysfunction is characterized by an imbalance between vasoconstrictor and vasodilator responses leading to impaired vascular tone, peripheral vascular resistance, and organ perfusion, and is one of the earliest events in the development of atherosclerotic lesions. Metabolic Syndrome is often associated with impaired endothelium-dependent vasodilation, likely due to insufficient NO production or bioavailability, [8] and with exaggerated vasoconstriction, due to an increased production of vasoconstricting mediators [9].

## 2. The Role of Berries in the Modulation of Metabolic Syndrome

Berries represent a variety of small fruits characterized by the red, purple, and blue color. The most common berries are: blueberry, bilberry, cranberry, blackberry, raspberry, black, white or red currant, and strawberry. Minor berries include: lingonberry, cloudberry, elderberry, honeyberry, whortleberry, and chokeberry.

Berries are consumed both as fresh product as well as processed foods (i.e., juices, beverages, jams, freeze-dried). They contain high levels of polyphenols including flavonoids (anthocyanins, flavonols, and flavanols), condensed tannins (proanthocyanidins), hydrolyzable tannins (ellagitannins and gallotannins), phenolic acids (hydroxybenzoic and hydroxycinnamic acids, chlorogenic acid), stilbenoids and lignans [10,11]. Their concentration varies according to species, genotype, growing and post-harvesting conditions [12].

Anthocyanins (ACNs) are probably the main bioactive compounds that characterize berries with pelargonidin, cyanidin, delphinidin, petunidin, peonidin, and malvidin the most predominant ACN compounds. They are found mainly in the external layer of the pericarp. ACNs include aglycones–anthocyanidins and their glycosides–anthocyanins. They differ with regard to the position and number of hydroxyl groups, degree of methylation, type and number of sugar molecules (mono-, di- or tri-glycosides), type of sugars (the most common sugars include glucose, galactose and arabinose) and type and number of aliphatic or aromatic acids (i.e., *p*-coumaric, caffeic, ferulic acid). Among berries, blackcurrants, black elderberries, blackberries and blueberries are particularly rich in ACNs (400 to 500 mg/100 g) [13]. Phenolic acids are represented by hydroxycinnamic acids (i.e., ferulic, caffeic, p-cumaric acids. and caffeoylquinic esters) and benzoic acid derivatives (i.e., gallic acid, salicylic, p-hydroxybenzoic. and ellagic acids). They occur mainly in bound forms as esters or glycosides. Gallic acid and chlorogenic acids are abundant in blueberry (~200 mg/100 g) and blackberry (~300 mg/100 g). Ellagic acid is the main phenolic acid in strawberries (from 300 mg to 600 mg/100 g) where it is present in free form or esterified to glucose in hydrolysable ellagitannins. Blueberries and cranberries are also important sources of ferulic acid, bilberries and blackcurrants of *p*-coumaric and caffeic acid, while chokeberries are rich sources of caffeic, chlorogenic, and neochlorogenic acids [13].

Flavonols, 3-hydroxyflavones and tannins are also widespread in berries. Flavan-3-ols are a complex subclass of polyphenols without glycoside residues and with different levels of polymerization ranging from monomeric, oligomeric, and polymeric forms. Tannins include both condensed non-hydrolysable tannins known as proanthocyanidins, and esters of gallic and ellagic acids called hydrolysable tannins. Tannins play an essential role in defining the sensory properties of fresh fruit and fruit-derived products. They are responsible for the taste and changes in the color of fruit and fruit juice. Moreover, tannins stabilize ACNs by binding to form co-polymers. The amount of flavan-3-ols and proanthocyanidins in chokeberries, blueberries, and strawberries varies from 150 mg to 700 mg/100 g, while in blackberries and raspberries the amount is about 300 mg/100 g [13].

Blackberries, blueberries, and strawberries are rich sources of ellagitannins (up to 600 mg/100 g), followed by chokeberries, cloudberries and red raspberries (~260 mg/100 g), while small quantities of tannins are found in honeyberries [13].

During recent years, a multitude of clinical research studies have focused on the health properties of berries. In particular, increasing attention has been devoted to the role of berries and their components in the modulation of oxidative stress [14], vascular function [15], inflammation, and lipid metabolism [16]. In addition, research studies have explored the role of berries on chronic diseases such as cardiovascular diseases, diabetes, and obesity with promising, albeit preliminary, results. Several studies have also investigated the effect of berry consumption on their ability to modulate/attenuate risk factors associated with Metabolic Syndrome.

**Figure 1:**
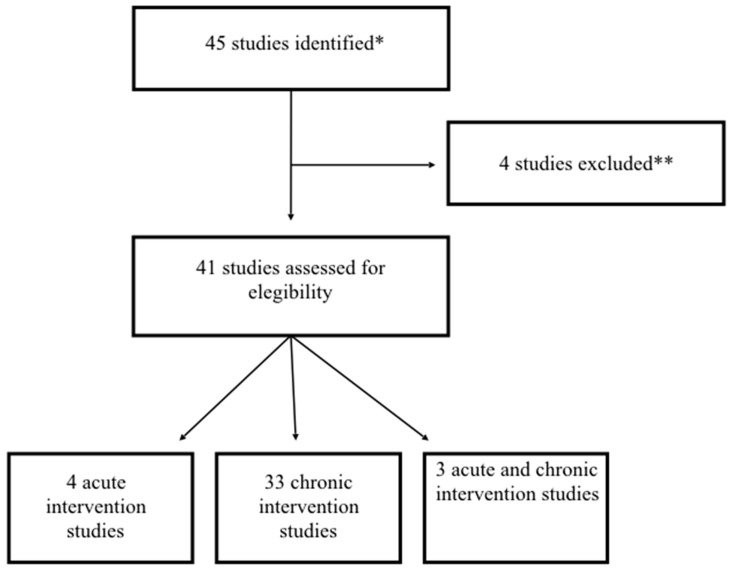
A flow chart highlighting study selection. * Studies were identified according to the following keywords: “berry”, “Metabolic Syndrome”, “overweight”, “obesity”, “hypertension”, “hypercholesterolemia”, “hyperlipemia”, “Type II diabetes”, and “humans”. ** Studies were excluded because they used a mix of fruit and vegetables and other foods in which berries were not the main food.

A search of the literature on intervention studies investigating the effect of berry consumption in the modulation of Metabolic Syndrome and/or related risk factors was carried out. Abstracts and full texts from human acute and chronic intervention studies were screened. PUBMED, ScienceDirect, and ScholarGoogle databases were searched to identify articles published later than 1 January 2000. The searches used the following terms and text words alone and in combination: “berry”, “Metabolic Syndrome”, “overweight”, “obesity”, “hypertension”, “hypercholesterolemia”, “hyperlipemia”, “Type II diabetes”, and ”humans”. Interventions conducted in healthy subjects (not presenting any of the risk factors characterizing Metabolic Syndrome) were excluded. Reference lists of the obtained articles were also searched for related articles. The search was limited to English-language articles. A total of 45 articles were obtained from the database searches and from their reference lists. Four papers were excluded because studies were performed with a mix of fruits and vegetables and other foods in which berries did not constitute the main food [17,18,19,20]. Therefore after exclusions, a total of 41 studies were included in the review (Figure 1). Thirty-four explored a medium-long term intervention, four were post-prandial while three investigated both or performed a chronic intervention followed by an acute study. Here, we summarize the main results of the human studies. The results obtained are also reported in Table 1, describing the type of food or supplement, number of intervention days, number of subjects and their characteristics, dose/day of test food, the use of control/placebo food, and the significant findings.

## 3. Blueberry

Depending on the size of the shrubs, blueberries are classified as either lowbush (‘wild’) or highbush: *Vaccinium corymbosum*, a highbush blueberry, is the most commonly cultivated species around the world, while *Vaccinum angustifolium*, native to North America, is the most widespread species of wild blueberry [62]. Anthocyanins are the distinctive and most abundant class of phenolic compounds in blueberries, and wild blueberries contain higher amounts of anthocyanins compared to highbush blueberries. Blueberries also contain significant amounts of phenolic acids, flavonols and flavan-3-ols, as well as manganese [63].

In an 8-week, controlled, randomized intervention, 48 middle-aged, obese subjects with Metabolic Syndrome were given a daily blueberry drink made with 50 g freeze-dried blueberries, resulting in significantly lower systolic and diastolic blood pressure, plasma oxidized LDLs, and malondialdehyde (MDA) and 4-hydroxynonenal (HNE) concentrations. No significant changes were observed for cholesterol and triglyceride levels, inflammatory markers CRP, adiponectin and IL-6, or adhesion molecules ICAM-1 and VCAM-1 [25].

In a 6-week, placebo-controlled, randomized intervention, 27 obese, insulin-resistant subjects received a daily smoothie made with 45 g of blueberry powder, which resulted in improved insulin sensitivity but did not affect markers of inflammation, blood pressure, and blood lipid profile [24]. In a subsequent 6-week intervention with the same experimental design on 44 obese subjects with Metabolic Syndrome, endothelial function measured as peripheral arterial tone, significantly improved with blueberry consumption, while blood pressure and insulin sensitivity did not significantly change [22].

In a 6-week, placebo-controlled, cross-over, randomized intervention on 18 subjects with risk factors for cardiovascular disease receiving a daily blueberry drink made of 25 g wild blueberry powder, endogenous and ex vivo oxidatively-induced DNA damage in blood mononuclear cells was found to be significantly lower, but no changes were observed in blood lipid profile, markers of inflammation, and endothelial function [23].

In an 8-week, placebo-controlled, cross-over, randomized intervention on 48 overweight or obese hypertensive women, administration of a daily drink made with 22 g of blueberry powder resulted in significantly lower systolic and diastolic blood pressure and brachial-ankle pulse wave velocity, and higher plasma levels of nitric oxide, but no changes in the inflammatory marker CRP [21].

In conclusion, the effect of blueberries in the modulation of Metabolic Syndrome-related risk factors has been investigated in five interventions, documenting an effect on blood pressure, oxidative stress, endothelial function, and insulin sensitivity, but not on inflammatory marker levels or blood lipid profile.

## 4. Bilberry

Bilberries (*Vaccinium myrtillus*), the wild European blueberries, have a phenolic profile and content which is comparable to that of blueberries, and are also exceptionally rich sources of anthocyanins.

In a 4-week, controlled, randomized intervention, 62 subjects with risk factors for cardiovascular disease received 330 mL/day of bilberry juice, resulting in decreased serum concentrations of inflammatory markers CRP, IL-6, IL-15 and TNF-α, while markers of antioxidant status and oxidative stress remained unaffected [28].

When 100 g/day of fresh bilberries were given to 80 overweight or obese women with or without Metabolic Syndrome for 5 weeks, body weight, waist circumference and concentrations of VCAM-1 and TNF-α, but also adiponectin, significantly decreased, insulin and glycated hemoglobin concentrations increased, while fat percent, blood pressure, blood lipids, and IL-6 levels were not affected [27].

In an 8-week, controlled, randomized intervention, in 27 overweight or obese subjects with Metabolic Syndrome receiving 200 g/day of fresh bilberry purée and 40 g/day of dried bilberries, serum concentrations of inflammatory markers hs-CRP, IL-6, IL-12, and LPS decreased, with no changes in blood glucose and lipid profile [26].

Thus, the effect of bilberries on Metabolic Syndrome has not been fully investigated. To our knowledge, only three interventions focused on this berry, finding a consistent effect on reducing inflammation levels.

## 5. Cranberry

Native to North America, cranberry (*Vaccinium macrocarpon*) is a rich source of different phenolic antioxidant compounds, primarily phenolic acids and flavonoids including anthocyanins, flavonols, and flavan-3-ols [33]. Cranberries also contain resveratrols and the anti-inflammatory acetylsalicylic acid [29].

A randomized, placebo-controlled intervention administering six daily capsules of cranberry powder (equivalent to 240 mL cranberry juice) for 12 weeks to a group of 27 subjects with type 2 diabetes, did not detect any significant effect on blood glucose levels, glycated hemoglobin HbA1c, triglyceride or cholesterol levels [40].

A 2-week intervention on 21 dyslipidemic men with abdominal obesity, reported improved total plasma antioxidant capacity, and decreased BMI and plasma oxidized LDL levels following daily consumption of 7 mL/kg BW of cranberry juice. Blood pressure, plasma lipid profile, and inflammatory markers were not significantly affected [39].

When the effects of three different doses (125, 250, and 500 mL/day) of cranberry juice administered sequentially for four weeks each, were tested on a group of 30 middle-aged men with abdominal obesity, a significant reduction in body weight, BMI, waist circumference, total/HDL-cholesterol ratio and apolipoprotein B, and a significant improvement in plasma total antioxidant capacity were reported after 250 mL and/or 500 mL consumption. A significant decrease in plasma nitrite and nitrate, and increase in HDL cholesterol were reported only after the highest dose. No significant effect was observed at the lowest dose, and no effect on total, LDL and VLDL cholesterol was observed at any dose [38].

The same experimental design was tested on 30 middle-aged abdominally obese men, nine of which with Metabolic Syndrome. HDL cholesterol and oxidized LDLs improved after 250 and 500 mL cranberry juice consumption, while systolic blood pressure and plasma ICAM-1 and VCAM-1 concentrations decreased only at the highest dose, with a stronger effect in the subset of subjects with Metabolic Syndrome. No significant effect was observed at the lowest dose, and no effect on cholesterol, triglycerides, diastolic blood pressure, and plasma E-selectin was observed at any dose [35].

When the post-prandial effect of a single-dose of sweetened cranberry juice was compared to unsweetened cranberry juice in a group of 12 obese subjects with type 2 diabetes, only unsweetened cranberry juice resulted in reduced plasma insulin levels and glycemic response, compared to both a sweetened cranberry juice and an unsweetened control drink [37].

With a similar experimental design, the post-prandial effects of either raw cranberries, sweetened dried cranberries or dried cranberries sweetened with less sugar were tested in 13 obese subjects with type 2 diabetes. The effects of raw cranberries were the lowest on glycemic and insulinemic responses, with the less sweetened, dried cranberries significantly lower compared to the fully sweetened ones [34].

In a 12-week, placebo-controlled, randomized intervention, 30 older overweight subjects with type 2 diabetes received a daily capsule of 500 mg cranberry powder extract, resulting in lower total and LDL cholesterol, and total/HDL-cholesterol ratio. Blood glucose, insulin, and glycated hemoglobin HbA1c levels were unaffected, as well as CRP, blood pressure, oxidized LDLs, triglycerides, and HDL-cholesterol [36].

In a placebo-controlled, randomized intervention, 31 obese women with Metabolic Syndrome were given 480 mL/day of cranberry juice for eight weeks, resulting in reduced circulating levels of oxidized LDLs, malondialdehyde (MDA), and 4-hydroxynonenal (HNE), and improved total plasma antioxidant capacity, while blood pressure, blood lipid and glucose levels, and inflammatory markers did not change [33].

When a single-dose of 480 mL cranberry juice was administered to 15 subjects with coronary artery disease, post-prandial endothelial function measured as brachial artery flow-mediated dilation, it significantly improved (Dohadwala et al., 2011). When the same serving of cranberry juice was given daily to a group of 44 subjects with coronary artery disease in a 4-week, placebo-controlled, cross-over intervention, central aortic stiffness, measured as carotid-femoral pulse wave velocity, significantly decreased, as well as HDL-cholesterol levels. However, no changes were reported for total and LDL cholesterol, triglycerides, CRP, ICAM-1, blood pressure, carotid-radial pulse wave velocity or brachial artery flow-mediated dilation [32].

In a 12-week, placebo-controlled, randomized intervention on 58 middle-aged, overweight or obese subjects with type 2 diabetes, receiving 240 mL/day of cranberry juice, blood glucose, ApoB and ApoA1 concentrations significantly decreased, PON-1 activity increased, while lipoprotein(a) concentrations were unaffected [31].

When 56 overweight or obese subjects with Metabolic Syndrome were given 700 mL/day of low-calorie cranberry juice for four weeks, serum folic acid levels significantly increased while serum homocysteine levels, lipid and protein oxidation significantly decreased. Inflammatory markers CRP, TNF-α, IL-1, and IL-6 did not change [30].

Thirty five abdominally obese men with or without Metabolic Syndrome were given 500 mL/day of low-calorie 27% cranberry juice for four weeks in a placebo-controlled, cross-over intervention. Measures of global endothelial function and arterial stiffness significantly decreased following cranberry consumption compared to baseline, but not compared to post-placebo (Ruel et al., 2013 [29]). Blood pressure and serum markers of endothelial function ICAM-a, VCAM-1 and E-selectin, as well as oxidized LDLs, did not change [29].

In summary, the effects of cranberries on markers of Metabolic Syndrome have been investigated in eleven interventions, resulting in a consistent effect on reducing oxidative stress, and some beneficial effects on BMI, blood cholesterol levels, and vascular function.

## 6. Raspberry

Black raspberry (Rubus occidentalis) is a flavonoid-rich member of the Rosaceae family common in Japan, China, and South Korea, where it is traditionally used to treat prostate and urinary diseases. It also contains significant amounts of tyrosol, resveratrol, tannins, and other phenolic acids [42].

In a 12-week, randomized, controlled intervention, a group of 77 Metabolic Syndrome patients received 750 mg/day of black raspberry powder in the form of capsules or a fiber- and sugar-matched control. Raspberry resulted in decreased total serum cholesterol and total/HDL-cholesterol ratio, with no other changes in serum lipid profile. Inflammatory markers IL-6 and TNF-α significantly decreased, and anti-inflammatory adiponectin significantly increased, while CRP, ICAM-1 and VCAM-1 were unaffected. Brachial artery flow-mediated dilation significantly improved [42].

In a controlled, cross-over intervention on a group of 10 older overweight or obese men, the short-term effect of relatively large amounts of lyophilized black raspberry powder (45 g/day for four days) was evaluated in countering the post-prandial inflammatory effects of a high fat meal. Serum IL-6 concentrations significantly decreased, but not TNF-α and CRP concentrations [41].

In conclusion, the impact of raspberry consumption on subjects with risk factor for Metabolic Syndrome has been evaluated in only two interventions, documenting some positive effects on blood cholesterol levels, inflammation and vascular function. Research is needed to investigate more fully the potential of this berry.

## 7. Chokeberry

Chokeberry (*Aronia melanocarpa*) is a violet-black, strongly-flavored berry, common in areas of North America and Eastern Europe. It is a rich source of polyphenols, particularly anthocyanins present in form of cyanidin-glucosides, but also caffeic acid, quercetin, procyanidins, and other flavonoids [46].

An 18-week intervention on a group of 58 overweight or obese men with mild hypercholesterolemia who were given 150 mL/day of chokeberry juice for two, 6-week long periods separated by a 6-week wash-out, resulted in decreased circulating levels of serum total and LDL cholesterol and increased serum HDL_2_ cholesterol. Furthermore, serum triglycerides, glucose, homocysteine, and fibrinogen levels also significantly decreased, as well as blood pressure, while inflammatory markers hs-CRP protein and lipid peroxides were not affected [46].

Eight-week administration of 300 mg/day of chokeberry extract to a group of 52 middle-aged individuals with Metabolic Syndrome, resulted in decreased levels of serum total and LDL cholesterol, with no changes in total HDL cholesterol. Serum triglycerides were also significantly decreased, as well as parameters of platelet aggregation and coagulation [45].

A 4-week intervention on 20 middle-aged, obese women with 100 mL/day of a glucomannan-enriched chokeberry juice resulted in decreased BMI, waist circumference and systolic blood pressure. However, no changes were observed in diastolic blood pressure, blood glucose levels, serum triglycerides, total and LDL cholesterol, with increased levels of HDL cholesterol. Membrane composition of erythrocytes was affected, with an increase in omega-3 polyunsaturated fatty acids and a decrease in monounsaturated fatty acids [44].

Four week administration of 200 mL/day of chokeberry juice to a group of 23 hypertensive subjects resulted in decreased systolic and diastolic blood pressure, but blood lipid profile, glucose and C-reactive protein levels were unaffected [43].

The effect of chokeberries on Metabolic Syndrome has been investigated in four intervention studies, documenting some positive but not consistent effects on blood cholesterol and triglyceride levels, blood pressure, and erythrocyte membrane fluidity. More research is needed to better investigate the effects of this berry.

## 8. Strawberry

Besides being an important source of vitamin C, strawberries (*Fragaria × ananassa*) contain an abundance of various phenolic compounds, including quercetin, ellagic acid, anthocyanins, catechins, and kaempferol [54].

Following a controlled 7-week, randomized, cross-over intervention, 28 hyperlipidemic subjects receiving a serving of strawberries (454 g/day) showed reduced LDL oxidative damage and a reduced LDL-cholesterol molar ratio compared to control. No significant effect was observed for plasma levels of conjugated dienes, lipid profile, C-reactive protein, blood pressure, and body weight [57].

An 8-week randomized, controlled trial on 27 subjects with Metabolic Syndrome receiving two cups of a strawberry beverage (composed of 25 g of freeze-dried strawberry powder each) daily for eight weeks, documented a reduction of total and LDL-cholesterol, small LDL particle size, and VCAM-1. No significant effect was observed for glucose, triglycerides, HDL-cholesterol, blood pressure, waist circumference, and ICAM-1 [55].

A 4-week intervention on 16 obese women with Metabolic Syndrome receiving two daily cups of a strawberry drink (composed of 25 g of freeze-dried strawberry powder each), reported a significant reduction in serum total and LDL-cholesterol levels, with no changes in HDL cholesterol, triglycerides, blood glucose levels and blood pressure. Lipid peroxidation products malondialdehyde (MDA) and 4-hydroxynonenal (HNE) significantly decreased, but not plasma oxidized LDL, and inflammatory markers hs-CRP and adiponectin [56].

A placebo-controlled, randomized, crossover 6-week intervention on 24 middle-aged, overweight or obese hyperlipidemic subjects receiving a daily strawberry drink (composed of 10 g of freeze-dried strawberry powder) did not find any differences in fasting triglycerides, cholesterol or plasma oxidized LDL concentrations. However, when the postprandial response to a high-fat meal challenge was evaluated at the end of the intervention, subjects who were on the strawberry group had significantly lower triglycerides as well as total, LDL and HDL-cholesterol levels [54].

A placebo-controlled, randomized, 6-week intervention on 24 overweight subjects receiving the same strawberry drink, found no changes in serum glucose, insulin, hs-CRP, IL-6, PAI-1, IL-1β, and TNF-α concentrations. However, when a high carbohydrate and fat meal was given to the subjects at the end of the intervention, serum PAI-1, IL-1β levels were significantly lower in the strawberry group, but not serum glucose, insulin, hs-CRP, IL-6, and TNF-α levels [53].

When the post-prandial effect of a single-dose of the same strawberry drink was evaluated on a group of 26 overweight individuals consuming a high-carbohydrate moderate-fat meal, without any pre-intervention, serum hs-CRP, IL-6 and insulin levels were found to be significantly lower, but not PAI-1, IL-1β, TNF-α, and blood glucose concentrations [52].

Following a controlled 7-week, randomized, cross-over intervention, 20 obese individuals receiving two servings of strawberry powder (equivalent to 320 g/day of frozen strawberries) showed a significant reduction in serum total and small HDL-cholesterol concentrations and in LDL particle size. No changes in serum triglycerides and inflammatory markers and oxidative stress were observed, but serum fibrinogen concentrations decreased [51].

When a group of 36 overweight subjects with type 2 diabetes received 50 g/day of freeze-dried strawberry for six weeks in a placebo-controlled, randomized intervention, markers of total serum antioxidant status significantly increased while serum malondialdehyde (MDA), glycated hemoglobin HbA1c, and hs-CRP concentrations significantly decreased, and blood glucose levels remained unaffected [50].

In another controlled, randomized intervention, 36 overweight or obese subjects with type 2 diabetes received the same amount of freeze-dried strawberry for six weeks, resulting in a significantly lower diastolic blood pressure, but with no changes in systolic blood pressure as well as serum triglycerides and cholesterol levels [49].

In a 12-week, randomized, controlled intervention, 60 obese volunteers with risk factors for cardiovascular disease were assigned to either a low-dose or high-dose strawberry treatment (25 or 50 g freeze-dried strawberry powder in water, respectively), or a calorie- and fiber-matched control. At the end of the intervention, malondialdehyde (MDA) and 4-hydroxynonenal (HNE) significantly decreased with both doses, but a significant reduction in serum total and LDL-cholesterol, and derived small LDL particles, was only observed in the high-dose group. Serum glucose, glycated hemoglobin HbA1c, insulin, HDL, VLDL-cholesterol, HOMA-IR score, triglycerides, VCAM-1, ICAM-1, and hs-CRP did not change significantly [48].

When a single serving of freeze-dried strawberry powder at different doses (0, 10, 20 or 40 g) was given to a group of 21 abdominally obese subjects with insulin resistance, a significant reduction in post-prandial plasma insulin concentrations, insulin:glucose ratio, and rate of glucose and insulin increase was only observed after consumption of the highest dose. Interestingly, a significant reduction in plasma oxidized LDLs was observed after the 20 g dose, but not after the 40 g dose. Plasma glucose, triglycerides and IL-6 levels did not change at any dose [47].

Thus, ten studies focused on the role of strawberries on Metabolic Syndrome and its risk factors. Overall, strawberry consumption led to some improvements in oxidative stress, inflammatory status, blood lipid profile, and blood pressure, although these effects were not consistent.

## 9. Whortleberry

Caucasian whortleberry, or qaraqat (*Vaccinium arctostaphylos*), is a wild berry, common in Western Asia with high anthocyanin content [58]. In a 4-week, placebo-controlled, randomized intervention on 50 hyperlipidemic subjects, two daily capsules of whortleberry extract containing 45 mg of anthocyanins each, resulted in lower serum concentrations of total and LDL cholesterol, triglycerides, and MDA, without changes in HDL-cholesterol and inflammatory marker hs-CRP [47]. Due to the paucity of data regarding this berry, more research is needed to investigate its effects.

## 10. Berry Mix

Considering the positive results obtained from single-berry studies, some research groups tested the effect of a mix of berries in randomized interventions.

In a 12-week, placebo-controlled study on 133 older, borderline or hypertensive subjects, consumption of 500 mL/day of a juice made from red grapes, cherries, chokeberries, and bilberries resulted in significantly lower systolic blood pressure after six, but not after 12 week consumption , and no effect on diastolic pressure. When only the hypertensive sub-group was considered, the blood pressure lowering effect was more pronounced [59].

In a 12 week intervention on 20 overweight or obese subjects with Metabolic Syndrome risk factors, daily combined consumption of 100 g strawberry purée, 100 g of frozen raspberries, and 100 g of frozen cloudberries, resulted in lower serum leptin levels but did not affect blood pressure, serum lipid profile, resistin, and markers of oxidative stress [60].

In a 20 week intervention on 61 overweight or obese women with Metabolic Syndrome, a daily mix of 163 g of lingonberry, sea buckthorn berry, bilberry, and black currant resulted in higher anti-inflammatory adiponectin plasma concentrations, but did not affect blood pressure, blood lipid or glucose levels, markers of oxidative stress, and other markers of inflammation [61].

More studies investigating the effects of a mix of multiple berries would be useful, since berries are often commercialized in mixes, both as fresh products but above all in juices, smoothies, purees, and other products.

## 11. Remarks and Conclusions

In the US, more than one in every five adults meets the diagnostic criteria for Metabolic Syndrome [64]. In turn, this condition dramatically increases the risk of developing type II diabetes and suffering acute cardiovascular events, such as heart attacks or strokes [1]. Thus, finding economic and effective ways to prevent and reverse Metabolic Syndrome is of key importance for public health.

Several lines of evidence suggest that diet, together with regular physical activity and avoidance of smoking, is one of the most manageable ways of preventing the development of Metabolic Syndrome in the human population, and at the same time it is also a tool to mitigate the symptoms and decrease the risk of complications in patients who already suffer from this condition.

Furthermore, diet has the potential to greatly reduce the need for pharmacological treatment, which is inevitably associated with harmful side effects and constitutes a considerable financial burden. Being a multifactorial condition, the pharmacological approach to Metabolic Syndrome requires the use of multiple medications, to control a wide array of metabolic abnormalities ranging from dyslipidemia to hypertension, and from hypercholesterolemia to impaired blood glucose control. Thus, the risk of side effects is multiplied by the need of administering multiple medications, and the opportunity to be able to prevent or even control some of the same metabolic dysfunctions with lifestyle—including diet—becomes especially advantageous.

Mounting evidence suggests that consumption of dietary achievable amounts of berries, whose distinctive nutritional characteristic is the abundance of phenolic compounds, has the potential to affect several metabolic abnormalities related to Metabolic Syndrome.

When results of the dietary interventions reviewed in this article are considered together, it appears that the stronger and most recurrent effects of berry consumption lie in their anti-inflammatory and anti-oxidant effects. They also have a tendency to improve lipid profile, lowering total and LDL-cholesterol as well as triglycerides, but this outcome is not observed consistently. It is reasonable to speculate that inter-individual variability as well as interactions with the rest of the diet, are strong enough to mask the effects of berry consumption on lipid profile, and this seems to be confirmed by the observation that effects on blood lipids tend to reach statistical significance in subjects with particularly abnormal baseline values.

Several studies also report positive effects in attenuating blood pressure, especially systolic blood pressure in hypertensive subjects, and some studies document positive effects on markers of endothelial function.

As far as the effects on glucose and insulin metabolism are concerned, results are mixed and tend to be absent, in a few studies even negative. It is possible that in subjects whose glucose and insulin metabolism is already severely impaired, the benefits of phenolic compounds are outweighed by the sugar content of berries, especially when given in form of juices and not with meals.

Another recurring observation in studies testing multiple doses is that there exists a dose-response effect, and some positive outcomes are only observed at higher levels of consumption or with longer interventions.

Considering the overall positive effects of berry consumption that have been reported in several dietary interventions on multiple metabolic abnormalities related to Metabolic Syndrome, it appears that regular berry consumption is a promising strategy to prevent Metabolic Syndrome and its complications, certainly not by itself as a single-bullet solution, but as part of a varied, balanced, and healthy dietary approach promoting health and preventing disease.

## Figures and Tables

**Table 1:** Effect of berry consumption on Metabolic Syndrome and associated risk factors.

Berry	Intervention	Participants	Dose	Main findings	References
Blueberry	8-week, randomized, double-blind, placebo-controlled, parallel intervention	Forty-eight postmenopausal women (Blueberry group: BMI 30.1 ± 5.94 kg/m^2^; age 59.7 ± 4.58 year; Control group: BMI 32.7 ± 6.79 kg/m^2^; age 57.3 ± 4.76 year) with pre- and stage 1-hypertension	Blueberry group: 480 mL blueberry drink (22 g freeze-dried blueberry powder corresponding to 1 fresh cup blueberries) Control group: 480 mL placebo drink	↓systolic, diastolic blood pressure and brachial-ankle pulse wave velocity ↑NO plasma levels ↑superoxide dismutase activity after blueberry and control group =weight, waist circumference, CRP	Johnson et al. [21]
	6-week randomized, double-blind, placebo-controlled, parallel intervention	Forty-four subjects with metabolic syndrome (Blueberry group: BMI 35.2 ± 0.8 kg/m^2^; age 55 ± 2 year; Control group: BMI 36.0 ± 1.1 kg/m^2^; age 59 ± 2 year)	Blueberry group: Smoothie prepared with 45 g blueberry powder Control group: identical smoothie without blueberry bioactives	↑endothelial function =blood pressure and insulin sensitivity	Stull et al. [22]
	6-week, randomized, placebo-controlled, crossover intervention	Eighteen male (BMI 24.8 ± 2.6 kg/m^2^; age 47.8 ± 9.7 year) with CVD risk factors	Wild blueberry group: 250 mL blueberry drink (25 g WB powder, equivalent to 148 g fresh WB) Control group: 250 mL water with sensory characteristics similar to the WB drink	↓Endogenous and oxidatively-induced DNA damage in PBMCs =lipid profile, weight, markers of inflammation and endothelial function, dietary markers, DNA repair activity	Riso et al. [23]
	6-week, randomized, double-blinded, placebo-controlled, parallel intervention	Twenty-seven (BMI between 32 and 45 kg/m^2^; age > 20 year) obese, insulin-resistant subjects	Blueberry group: smoothie prepared with 45 g of blueberry powder (22.5 g twice a day) (equivalent to 2 cups of fresh blueberries) Control group: identical smoothie without blueberry bioactives	↑insulin sensitivity =markers of inflammation, lipid profile and blood pressure	Stull et al. [24]
	8-week, randomized, single-blinded, controlled parallel intervention	Forty-eight subjects with metabolic syndrome (4 males and 44 females; BMI 37.8 ± 2.3 kg/m^2^; age 50.0 ± 3.0 year)	Blueberry group: 480 mL blueberry drink (50 g freeze-dried blueberries corresponding to 350 g fresh berries) Control group: 480 mL water	↓systolic and diastolic blood pressure ↓plasma ox-LDL, MDA and HNE levels =lipid profile, weight, waist circumference, inflammation markers	Basu et al. [25]
Bilberry	8-week, randomized, controlled, parallel intervention	Twenty-seven subjects (Bilberry group: BMI 31.4 ± 4.7 kg/m^2^; age 53 ± 6 year; Control group: 32.9 ± 3.4 year; age 50 ± 7 year) with metabolic syndrome	Bilberry group: 200 g of bilberry purée and 40 g of dried bilberries (eq. 200 g of fresh bilberries) Control group: habitual diet. The use of berries was allowed at maximum of 1 dL/day (corresponding to 80 g/day).	↓serum levels of hs-CRP, IL-6, IL-12 and inflammation score. ↓expression of MMD and CCR2 transcripts associated with monocyte and macrophage function associated genes =body weight, glucose, and lipid profile	Kolehmainen et al. [26]
	5-week, randomized, cross-over intervention	Eighty overweight and obese women (BMI 29.6 ± 2.1 kg/m^2^; age 44.2 ± 6.2 year; 21 subjects meeting metabolic syndrome criteria)	Bilberry group: 100 g fresh bilberries Sea buckthorn group: Sea buckthorn (SB), SB fractions or SB oils (equivalent to 100 g of berries) Control group: none	↓body weight, waist circumference, VCAM-1, TNF-α, adiponectin ↑insulin, GHbA_1_C =fat percent, blood pressure, fasting plasma cholesterol, triacylglycerol, ALAT and IL-6 serum levels	Lehtonen et al. [27]
	4-week, randomized, controlled, parallel intervention	Sixty-two subjects (Bilberry group: BMI range 19.9–31.7 kg/m^2^; age range 34–68 year; Control group: BMI range 17.8–31.5 kg/m^2^; age range 30–68 year) with CVD risk factors	Bilberry group: 330 mL bilberry juice/day (diluted to 1 L using tap water) Control group: 1 L of water	↓serum levels of CRP, IL-6, IL-15, TNF-α, MIG =markers of antioxidants status and oxidative stress	Karlsen et al. [28]
Cranberry	4-week, placebo-controlled double-blind, crossover intervention	Thirty-five abdominally obese men (age 45 ± 10 year, BMI 28.3 ± 2.4 kg/m^2^ and a waist circumference ≥90 cm) with metabolic (*n* = 13) and without metabolic syndrome (*n* = 22)	Cranberry group: 500 mL/day of either low-calorie juice (27% juice) Control group: 500 mL/day placebo juice	↓arterial stiffness and global endothelial function =blood pressure, markers of endothelial function	Ruel et al. [29]
	60 days, parallel intervention	Fifty-six subjects (cranberry group: BMI 30.9 kg/m^2^ median, age 51.0 year median; control group: BMI 34.0 kg/m^2^ median, age 48.5 year median) with metabolic syndrome	Cranberry group: 700 mL/day reduced-energy cranberry juice Control group: usual diet	↓serum homocysteine levels, lipoperoxidation, protein oxidation ↑serum folic acid levels =metabolic and inflammatory biomarkers C-reactive protein, TNF-α, IL-1 and IL-6	Simão et al. [30]
	12-week, randomized, double-blind, parallel intervention	Fifty-eight (BMI 28.8 ± 3.6 kg/m^2^, age 54.8 ± 9.1 year) Type II diabetic subjects	Cranberry group: 240 mL/day cranberry juice Control group: 240 mL/day placebo juice	↓glucose, ApoB ↑ApoA-1, PON-1 activity =lipoprotein(a)	Shidfar et al. [31]
	Post-prandial 4-week, randomized, placebo-controlled, cross-over intervention	Fifteen (BMI not reported, age 62 ± 8 year, 13% female) subjects with coronary artery diseases Forty-seven subjects with coronary artery diseases (cranberry group: BMI 30 ± 5 kg/m^2^, age 61 ± 11 year; Control group: BMI 29 ± 4 kg/m^2^, age 63 ± 9 year)	Cranberry group: 480 mL cranberry juice Control group: None Cranberry group: 480 mL/day cranberry juice Control group: 480 mL/day placebo juice	↑flow mediated dilation and lnPAT score as markers of endothelial function =blood pressure, heart rate, brachial diameter, hyperemic flow ↓carotid-femoral pulse wave velocity (a measure of central aortic stiffness). and HDL-cholesterol after cranberry juice =lipid profile, glucose, insulin, HOMA-IR, C-reactive protein, ICAM-1 serum levels, brachial artery flow-mediated dilation, digital pulse amplitude tonometry, blood pressure, and carotid-radial pulse wave velocity	Dohadwala et al. [32]
	8-week, randomized double-blind, placebo-controlled, parallel intervention	Thirty-one (BMI 40.0 ± 7.7 kg/m^2^, age 52.0 ± 8.0 year) female with metabolic syndrome	Cranberry group: 480 mL/day cranberry juice Control group: 480 mL/day placebo drink	↓ox-LDL, MDA & HNE plasma/serum levels ↑Total plasma antioxidant capacity =blood pressure, glucose, plasma lipoprotein-lipid, markers of inflammation	Basu et al. [33]
	Post-prandial cross-over intervention	Thirteen (6 female and 7 male) noninsulin-dependent subjects (age 61.6 ± 2.3 year, BMI 33.25 ± 1.22 kg/m^2^)	Cranberry group: *Group 1*: raw cranberry (55 g, 21 cal, 1 g fiber) *Group 2:* sweetened dried cranberry (40 g, 138 cal; 2.1 g fiber) *Group 3:* sweetened dried cranberries-less sugars group (40 g; 113 cal; 1.8 g fiber + 10 g polydextrose) Control group: white bread (57 g, 160 cal; 1 g fiber)	↓glycemic and insulinemic response following SDC-LS	Wilson et al. [34]
	12-week intervention (three 4-week intervention with 125, 250 and 500 mL/day cranberry juice)	Thirty (BMI 27.8 ± 3.2 kg/m^2^, age 51 ± 10 year; 9 subjects with metabolic syndrome and 21 without metabolic syndrome) abdominally obese men	Cranberry group: 125, 250 and 500 mL/day cranberry juice Control group: none	↓ox-LDL following 250 and 500 mL cranberry juice ↓systolic blood pressure, s-VCAM, ICAM plasma levels following 500 mL cranberry juice ↓ox-LDL, ICAM plasma levels in subjects with metabolic syndrome following 12-week intervention ↑HDL cholesterol following 250 and 500 mL cranberry juice =Total, LDL Apo B cholesterol, triglycerides, diastolic blood pressure, heart beat, E-selectin plasma levels	Ruel et al. [35]
	12-week, randomized, placebo-controlled, double-blind, parallel intervention	Thirty (16 males and 14 females) Type II diabetic subjects (Cranberry group: 9/6 male/female, BMI 26.2 ± 0.7 kg/m^2^, age 65 ± 2 year; Control group: 7/8 male/female, BMI 25.9 ± 1.0 kg/m^2^, age 66 ± 2 year)	Cranberry group: 500 mg/capsule cranberry powder extract, three times a day Control group: 500 mg/capsule placebo	↓Total cholesterol, Total: HDL cholesterol ratio, LDL cholesterol =waist circumference, BMI, fasting serum glucose, insulin, HbA1c, HOMA insulin resistance, C-reactive protein, blood pressure, ox-LDL, triglyceride, HDL-cholesterol levels, uric acid	Lee et al. [36]
	Post-prandial intervention	Twelve (6 male and 6 female) type II diabetic subjects (age 65.3 ± 2.3 year, BMI 34.7 ± 1.6 kg/m^2^	Cranberry group: *Group 1:* normal calorie cranberry juice (NCCBJ; 27% cranberry juice, *v*/*v*; 130 Cal/240 mL) *Group 2:* unsweetened low-calorie cranberry juice (LCCBJ; 27%, *v*/*v* CBJ; 19 Cal/240 mL) Control group: *Group 1:* normal calorie control (NCC; 140 Cal/240 mL) made with dextrose *Group 2:* low-calorie control (LCC; 19 Cal/240 mL)	↓plasma insulin and glycemic response following LCCBJ	Wilson et al. [37]
	12-week intervention (three 4-week intervention with 125, 250, and 500 mL/day cranberry juice)	Thirty (BMI 27.8 ± 3.3 kg/m^2^, age 51 ± 10 year) abdominally obese men	Cranberry group: 125, 250 and 500 mL/day cranberry juice Control group: none	↓body weight, BMI, waist circumference, waist-to-hip ratio, total:HDL cholesterol apo B, after intervention with 250 and 500 mL cranberry juice ↑plasma nitrite/nitrate following intervention with 500 mL ↑plasma antioxidant capacity following 250 and 500 mL cranberry juice ↑HDL cholesterol following 250 mL cranberry juice =Total, LDL and VLDL cholesterol	Ruel et al. [38]
	2-week intervention	Twenty-one (BMI 26.9 ± 3.8 kg/m^2^, age 38 ± 8 year) abdominally obese-dyslipidemic men	Cranberry group: 7 mL/kg BW (range 460–760 mL/day cranberry juice) Control group: none	↓BMI, plasma ox-LDL levels ↑Total plasma antioxidant capacity =waist and hip circumference, waist/hip ratio, blood pressure, plasma lipoprotein-lipid, inflammation markers	Ruel et al. [39]
	12-week randomized, controlled, parallel intervention	Twenty-seven Type II diabetic subjects (cranberry group: 14 subjects, 6 women and 8 men, BMI not reported, age 57.9 ± 10.6 year; placebo group: 13 subjects 6 women and 7 men, BMI not reported, age 52.6 ± 13.7 year)	Cranberry group: 6 capsules (equivalent to 240 mL cranberry juice) containing cranberry juice concentrate powder Control group: 6 capsules containing placebo powder	↑insulin levels after placebo treatment =fasting serum glucose, HbA1c, fructosamine, triglyceride, HDL, LDL-cholesterol levels	Chambers & Camire [40]
Raspberry	14-day (4-day black-raspberry intake, post-prandial, randomized, cross-over intervention, wash-out and 4-day black-raspberry intake)	Ten older overweight and obese (BMI, 31.4 ± 2.7 kg/m^2^, age 64.7 ± 6.9 year) males	Berry group: 45 g/day of lyophilized black raspberry powder for 4 days + high fat meal post prandial Control group: No black raspberry + high fat meal post prandial	↓serum IL-6 levels =TNF-α, CRP	Sardo et al. [41]
	12-week, randomized, controlled, parallel intervention	Seventy-seven subjects (berry group: BMI, 26.3 ± 4.3 kg/m^2^, age 58.0 ± 9.2 year; control group: BMI, 25.1 ± 4.0 kg/m^2^, age 60.1 ± 9.5 year) with metabolic syndrome	Berry group: 750 mg/day of black raspberry powder as capsules Control group: 750 mg/day of cellulose, isomalto, and corn powder as capsules	↓total cholesterol level total cholesterol/HDL ratio IL-6, TNF-α ↑flow mediated dilation, adiponectin =serum lipid profile, CRP, ICAM and VCAM	Jeong et al. [42]
Chokeberry	4-week intervention	Twenty-three subjects (BMI, not reported, age 47.5 ± 10.4 year) with hypertension	Berry group: 200 mL/day of polyphenol-rich organic chokeberry juice Control group: none	↓systolic and diastolic blood pressure, heart rate high-frequency power, heart rate very low frequency, standard deviation of normal RR intervals Holter ECG =lipid profile, glucose, CRP, urea, creatinine, Ac. uricum, AST, ALT, markers related to short term heart rate and Holter ECG	Kardum et al. [43]
	4-week intervention	Twenty women (BMI, 36.1 ± 4.4 kg/m^2^; age 53.0 ± 5.4 year) with abdominal obesity	Berry group: 100 mL/day glucomannan-enriched (2 g), chokeberry juice-based Control group: none	↓BMI, waist circumference, systolic blood pressure, serum HDL cholesterol, erythrocytes monounsaturated fatty acids, n6/n3 ratio ↑erythrocytes n3 polyunsaturated fatty acids =erythrocytes saturated and n6 polyunsaturated fatty acids, unsaturation index, diastolic blood pressure, lipid profile, glucose and enzymatic activity (SOD, CAT, GPx)	Kardum et al. [44]
	8-week intervention	Fifty-two subjects (42–65 years old; berry group: 38 subjects, BMI 31.1 ± 3.3 kg/m^2^; control group: 14 healthy subjects; BMI 24.4 ± 1.5 kg/m^2^) with metabolic syndrome	Berry group: 300 mg/day chokeberry extract Control group: No intervention	↓serum total and LDL cholesterol, triglycerides, coagulation and platelet aggregation parameters =BMI, waist circumference, serum HDL cholesterol	Sikora et al. [45]
	18-week (6-week intervention + 6-week wash-out + 6 week intervention) intervention	Fifty-eight men (BMI 27.7 ± 2.9 kg/m^2^; age 54.1 ± 5.6 year) with mild hypercholesterolemia	Berry group: 150 mL/day chokeberry juice Control group: none	↓serum total and LDL cholesterol, triglycerides, glucose, homocysteine and fibrinogen, blood pressure ↑serum HDL_2_ cholesterol =serum HDL and HDL3 cholesterol, hs-CRP, lipid peroxides, uric acid	Skoczyñska et al. [46]
Strawberry	Post-prandial, randomized, controlled, 4-arm, crossover intervention	Twenty-one (BMI 40.2 ± 7.2 kg/m^2^; age 39.8 ± 13.8 year) subjects with abdominal obesity and insulin-resistance	Berry group: *Group 1:* 10 g freeze-dried whole strawberry powder FDS *Group 2:* 20 g FDS *Group 3:* 40 g FDS Control group: 0 g FDS	↓plasma insulin, insulin: incremental increase, glucose: incremental increase, insulin: glucose ratio after 40 g FDS consumption =plasma glucose, TG, ox-LDL, IL-6	Park et al. [47]
	12-week, randomized, controlled, parallel intervention	Sixty volunteers with CVD risk factors LD-FDS: 15 subjects (14 females/1 male; BMI, 34.5 ± 4.4 kg/m^2^; age, 50 ± 10 year) HD-FDS: 15 subjects (13 females/2 males; BMI, 38.0 ± 7.1 kg/m^2^; age, 49 ± 11 year) LD-C: 15 subjects (14 females/1 male; BMI, 37.0 ± 4.4 kg/m^2^; age, 48 ± 10 year) HD-C: 15 subjects (14 females/2 male; BMI, 35.0 ± 5.2 kg/m^2^; age 48 ± 10 year)	Berry group: *Group 1:* low dose freeze-dried strawberries (LD-FDS): 25 g of freeze-dried powder reconstituted in 2 cups (474 mL/day) of water daily (corresponding to 250 g of fresh strawberries); *Group 2:* high dose freeze-dried strawberries (HD-FDS): 50 g of freeze-dried powder reconstitute in 2 cups (474 mL/day) of water (corresponding to 500 g of fresh strawberries) Control group: *Group 1:* Low-dose calorie- and fiber-matched control (LD-C) *Group 2:* high-dose calorie- and fiber-matched control (HD-C)	↓serum total and LDL-cholesterol, derived small LDL particles, MDA and HNE levels following HD-FDS intervention. ↓MDA and HNE following LD-FDS intervention =serum glucose, HbA1c, insulin, HDL, VLDL-cholesterol, HOMA-IR, TG, VACM-1, ICAM-1, hs-CRP	Basu et al. [48]
	6-week, randomized, double-blind, controlled, parallel intervention	Thirty-six diabetic subjects (berry group: *n* = 19; BMI, 27.36 ± 4.23 kg/m^2^; age 51.9 ± 8.2 year; control group: *n* = 17; BMI, 28.58 ± 4.7 kg/m^2^; age 51.1 ± 13.8 year)	Berry group: 2 cups freeze-dried strawberry (50 g/day) Control group: 2 cups iso-caloric drink with strawberry flavoring	↓diastolic blood pressure =serum TG, total cholesterol, ratio total cholesterol/HDL-cholesterol, systolic blood pressure, anthropometric indices	Amani et al. [49]
	6-week, randomized double-blind controlled intervention	Thirty-six subjects (23 females/13 males) with type 2 diabetes (berry group: 19 diabetic subjects; BMI, 27.32 ± 3.26 kg/m^2^; age, 51.88 ± 8.26 year; control group: 17 diabetic subjects; BMI, 28.70 ± 4.24 kg/m^2^, age, 51.17 ± 13.88 year)	Berry group: 50 g/day freeze-dried strawberry (equivalent to 500 g fresh strawberries) Control group: Placebo powder	↓MDA, HbA1c and hs-CRP serum levels ↑total serum antioxidant status =serum glucose levels	Moazen et al. [50]
	7-week double-blind, randomized, cross-over intervention	Twenty obese subjects (BMI between 30 and 40 kg/m^2^, age between 20 and 50 year)	Berry group: Strawberry powder (amount no reported) equivalent to 320 g/day of frozen strawberries. Two servings of strawberry powder mixed as a milkshake, in yogurt, cream cheese, or water-based, sweetened beverage Control group: Milkshake, yogurt, cream cheese, or water-based, sweetened beverage	↓Serum total cholesterol, small HDL particles, LDL size, fibrinogen =serum lipid profile, lipid particle concentrations and size, inflammatory markers and oxidative stress	Zunino et al. [51]
	Post-prandial, randomized, single-blind, placebo-controlled, cross-over intervention	Twenty-six overweight subjects (BMI, 29.2 ± 2.3 kg/m^2^; age, 50.9 ± 15.0 year)	Berry group: High-carbohydrate, moderate-fat meal + strawberry drink (10 g strawberry powder) Control group: High-carbohydrate, moderate-fat meal + placebo drink	↓serum hs-CRP, IL-6 and insulin levels =serum levels of PAI-1, IL-1β, TNF-α, glucose	Ederisinghe et al. [52]
	6-week, randomized, single-blind, placebo-controlled, parallel intervention + post-prandial high carbohydrates fat meal	Twenty-four overweight and obese subjects (BMI, 29.2 ± 2.3 kg/m^2^; age, 50.9 ± 15.0 year)	Berry group: 10 g/day strawberry powder in 305 mL water Control group: 10 g/day placebo powder in 305 mL water	=serum levels of glucose, insulin, hs-CRP, IL-6, PAI-1, IL-1β, TNF-α after 6-week intervention ↓serum PAI-1, IL-1β levels following 6-week + post-prandial intervention =serum levels of glucose, insulin, hs-CRP, IL-6, TNF-α after 6-week + post-prandial intervention	Ellis et al. [53]
	6-week, randomized, single-blind, placebo-controlled, crossover intervention After 6 weeks subjects consumed a high fat meal (post-prandial)	Twenty-four hyperlipidemic subjects (14 female, 10 male; BMI, 29.2 ± 2.3 kg/m^2^, age 50.9 ± 15 year)	Berry group: Drinks containing 10 g/serving of freeze-dried strawberry (equivalent to 110 g/day of fresh strawberries) Control group: Placebo drink	↓serum total, LDL and HDL cholesterol, TG after 6 weeks =ox-LDL plasma levels after 6 weeks ↓LDL and HDL cholesterol, TG and ox-LDL plasma levels after post-prandial compared to control group =serum total cholesterol after post-prandial	Burton-Freeman et al. [54]
	8-week intervention	Twenty-seven subjects (BMI: 37.5 ± 2.15 kg/m^2^; age: 47.0 ± 3.0 year) with metabolic syndrome	Berry group: Four cups of strawberry drink per day (each cup containing 25 g of freeze-dried strawberry powder) Control group: Four cups of water	↓total and LDL-cholesterol, small LDL particles, VCAM-1 =glucose, triglycerides, HDL-cholesterol, lipoprotein particle size and concentrations, blood pressure, and waist circumference, ICAM-1.	Basu et al. [55]
	4-week intervention	Sixteen female (mean BMI, 38.6 ± 2.3 kg/m^2^; range age, 39–71 year) with metabolic syndrome	Berry group: Two cups of strawberry drink per day (each cup containing 25 g of freeze-dried strawberry powder) Control group: None	↓serum total and LDL-cholesterol, MDA and HNE levels =serum HDL, TG, HDL, VLDL-cholesterol, hs-CRP, adiponectin, glucose, ox-LDL plasma levels, blood pressure, body weight, waist circumference	Basu et al. [56]
	4-week, randomized, controlled, cross-over intervention	Twenty-eight hyperlipidemic subjects (range BMI, 19.8–32.3 kg/m^2^; range age, 38–75 years)	Berry group: 454 g/day strawberries Control group: 65 g/day oat bran bread	↓MDA plasma absolute concentration and molar ration of LDL-cholesterol at 4 weeks compared to placebo ↑protein thiols concentration for both the treatments compared to each baseline =plasma levels of conjugated dienes, lipid profile, C-reactive protein, blood pressure, body weight	Jenkins et al. [57]
Whortleberry	4-week, randomized, double-blind, placebo-controlled, parallel intervention	Fifty hyperlipidemic subjects (berry group: twenty-five, 15 females/10 males; BMI, 25.40 ± 1.75 kg/m^2^; age, 48.08 ± 16.39 year; control group: twenty-five hyperlipidemic subjects, 15 females/10 males; BMI, 25.21 ± 2.01 kg/m^2^; age, 46.36 ± 16.59 year)	Berry group: 2 capsules/day whortleberry capsules Control group: 2 capsules/day placebo capsules	↓serum total, LDL-cholesterol, TG and plasma MDA levels =serum HDL-cholesterol levels, hs-CRP and BMI	Soltani et al. [58]
Berry mix	12-week, randomized, double-blinded, placebo-controlled intervention	One hundred and thirty-three hypertensive subjects (BMI, 26 ± 3 kg/m^2^, age 62 ± 6 year)	1 Berry group: 500 mL/day (MANA Blue juice: red grapes, cherries, chokeberries and bilberries) 2 Berry group: 500 mL/day (Optijuice: MANA + polyphenol-rich extract from blackcurrant press-residue) 3 Control group: 500 mL/day placebo	↓blood pressure	Tjelle et al. [59]
	12-week, randomized, controlled, parallel intervention	Twenty subjects (berry group: BMI, 31.8 ± 4.4 kg/m^2^, age 53.0 ± 6.5 year; control group: BMI, 32.9 ± 3.4 kg/m^2^, age 49.8 ± 7.1 year) with symptoms of metabolic syndrome	Berry group: 300 g/day fresh berries comprising of 100 g of strawberry purée, 100 g of frozen raspberries, and 100 g of frozen cloudberries Control group: Usual diet	↓serum leptin levels ↑microbiota in both the groups =blood pressure, serum lipid profile, 8-isoprostanes, resistin, TRAP assay	Puupponen-Pimiä et al. [60]
	20-week, randomized, controlled, parallel intervention	Sixty-one female subjects 35–52 years (berry group: BMI 29.3 ± 2.2 kg/m^2^, control group: BMI 29.5 ± 1.8 kg/m^2^) with metabolic syndrome	Berry group: 163 g/day mix of northern berries (lingonberry, sea buckthorn berry, bilberry and black currant) Control group: Usual diet	↓plasma ALT levels ↑plasma adiponectin levels =blood pressure, lipid profile, glucose, inflammatory markers, antioxidant capacity	Lehtonen et al. [61]

*Legend:* BMI: body mass index; NO: nitric oxide; hs-CRP: high sensitive C-reactive protein; ICAM: Intercellular Adhesion Molecule 1; VCAM: Vascular cell adhesion molecule 1; ALT/ALAT: alanine aminotransferase; TRAP: total radical trapping antioxidant parameter; TG: triglycerides; LDL: low density lipoprotein; ox-LDL: oxidized low density lipoprotein; HDL: high density lipoprotein; MDA: malondialdehyde; PBMC: peripheral blood mononuclear cell; HNE: 4-hydroxynonenal; IL-1β: interleukin-1β; IL-6: interleukin-6; TNF-α:tumor necrosis factor-alpha; PAI-1: plasminogen activator inhibitor-1; HOMA-IR: Insulin resistance index; GST: glutathione S-transferase; SOD: superoxide dismutase; GPx: glutathione peroxidase; GHbA**_1_**C: Glycated hemoglobin A_1_C; PAT: peripheral arterial function; PON1: paraxonase1; MIG: monokine induced by interferon; MMD: monocyte to macrophage differentiation associated; CCR2: chemokine (C-C motif) receptor 2.
